# Current Advances in Retroviral Gene Therapy

**DOI:** 10.2174/156652311795684740

**Published:** 2011-06

**Authors:** Youngsuk Yi, Moon Jong Noh, Kwan Hee Lee

**Affiliations:** 1 TissueGene Inc., Rockville, MD 20850, College of Medicine, Inha University, Inchon, South Korea 400-711; 2 Clinical Research Center, College of Medicine, Inha University, Inchon, South Korea 400-711

**Keywords:** Retroviral, Gene, Therapy, Safety, Insertional, Targeted, Vectors, Insulator.

## Abstract

There have been major changes since the incidents of leukemia development in X-SCID patients after the treatments using retroviral gene therapy. Due to the risk of oncogenesis caused by retroviral insertional activation of host genes, most of the efforts focused on the lentiviral therapies. However, a relative clonal dominance was detected in a patient with β-thalassemia Major, two years after the subject received genetically modified hematopoietic stem cells using lentiviral vectors. This disappointing result of the recent clinical trial using lentiviral vector tells us that the current and most advanced vector systems does not have enough safety. In this review, various safety features that have been tried for the retroviral gene therapy are introduced and the possible new ways of improvements are discussed. Additional feature of chromatin insulators, co-transduction of a suicidal gene under the control of an inducible promoter, conditional expression of the transgene only in appropriate target cells, targeted transduction, cell type-specific expression, targeted local administration, splitting of the viral genome, and site specific insertion of retroviral vector are discussed here.

## INTRODUCTION

Retroviral vectors have been the most preferred gene transfer systems in clinical gene therapy until the incident of a human trial for the X-linked severe combined immunodeficiency (SCID) [1-3]. The potential risk of insertional oncogenesis was realized in the trial, infants with X-SCID were cured by retrovirus-mediated *ex-vivo* gene transfer, and the trial was credited as the first unequivocal success for gene therapy [4]. However, four out of nine successfully treated patients later developed leukemia, and it is generally believed that leukemeogenesis was triggered by unexpected activation of a cellular proto-oncogene as a result of retroviral integration. Since our last review about the safety issues related to the use of retroviral vectors in human clinical trials, additional leukemeogenesis were reported [5-7]. It is time to re-evaluate all the safety issues concerning the use of retroviral vectors.

Stable incorporation of retroviral viral DNA into the host genome is in itself advantageous, as long-term expression of the transgene is possible for achieving therapeutic efficacy. However, a non-specific incorporation of viral DNA throughout the host genome can either cause a disruption of a host gene at the site of incorporation or cause an abnormal expression of nearby host genes driven by the enhancer of the inserted viral DNA. An insertional interference occurs against a host gene involved in a critical cellular function such as cell cycle progression, can become a major cause of cell transformation and oncogenesis, especially in the presence of additional physiological or genetic insults.

In addition to the risk of insertional mutagenesis, another safety issue with the retroviral vector is the possibility of generating replication competent retroviruses (RCR). Although new generations of retroviral vectors are designed to reduce the production of RCR, additional efforts are required to ensure the complete elimination of the problem.

Recently developed lentiviral gene transfer systems share many features of the retroviral systems. The viral genome integrates into host chromosomes, and inserted genes are maintained in the cells permanently. In contrast to conventional retroviral vectors, which require cell division for infection, lentiviral vectors infect efficiently non-dividing cells as well as dividing cells. Therefore, lentiviral vectors can be applied for transgene expression in neuronal cells. Most of the lentiviral vectors used in gene therapy are based on the human immunodeficiency virus (HIV). The major limitations of using HIV-originated lentiviral vectors in clinical trial are the safety concerns related to their HIV origin. Recent trials of lentiviral vector systems will be described in relevant chapters.

In this review, we will discuss about safety issues relating to the use of retroviral vectors for the therapeutic gene delivery. Suggestions for the possible solutions to these issues and future directions for an overall increase in the safety and efficiency of retroviral gene therapy protocols will be provided. Other important parameters in cell-based *ex-vivo* retroviral gene therapy protocols are already covered elsewhere [8], including cell type and numbers, observation period, proliferation capacity, age of patients, immunity, and side effects of transgene expression.

## TARGETED RETROVIRAL TRANSDUCTION

Current retroviral transfer system lacks specificity for target cell types. Non-specific infection hinders the application of retroviral system for the gene transfer to particular cell types in a mixed cell population. Tissue targeting is highly desirable and is expected to be valuable for various *in vivo* gene therapy protocols [9].

Infection of target cells by retroviruses is initiated by binding of the viral envelope protein to cell surface receptors [10]. Infusion of viral and cellular membranes leads to the internalization of the viral core [11]. In the past years, various retrovirus receptors, coreceptors and cofactors have been identified and studied for their role in viral entry [12], and attempts have been made to engineer viral envelope proteins and cellular receptors for attaining changes in the viral tropism [13, 14].

In an approach for a targeted transduction, envelope protein modification by attaching a peptide ligand such as epidermal growth factor (EGF) receptor binding domain to the NH_2_-terminus of the envelope glycoprotein (SU) was attempted [15-17]. Incorporation of the chimeric envelope protein into the viral particle allows binding of retrovirus to the receptor-positive target cells. The subsequent viral entry steps are blocked, however, because EGF receptors do not support these processes. As a protease cleavable linker was used in this chimeric protein to join the peptide ligand and SU, an appropriate cellular protease can cleave the attached ligand. Factor Xa [18, 19], plasmin [17], matrix-metallo-proteases (MMPs) [16], or intracellular protein convertases [20] were used for this purpose. Because overexpression of MMPs is frequently associated with angiogenesis, inflammation, and cancer invasion, MMPs are considered to be interesting targets for the protease-activatable gene delivery systems. Using MMP-activatable retroviral vectors, selective transduction of MMP-rich tumor cells was achieved in a heterogeneous cell population, but with somewhat reduced efficiency of transduction [17, 21]. Similarly, targeted infection for the high-molecular-weight melanoma-associated antigen (HMWMAA) expressing tumors was achieved by fusing a single chain antibody recognizing HMWMAA to the amino terminus of the surface domain of MLV with a matrix metalloprotease-2 (MMP2) cleavage site linker [22].

Matrix targeting is another approach and matrix-targeted retroviral vectors were found to be more efficient than unmodified vectors [23, 24]. A matrix-targeted retroviral vector was constructed by attaching collagen-binding polypeptide sequence to the amino-terminal region of the amphotropic 4070A envelope protein. Because tumor development and accompanied angiogenesis is associated with remodeling of extracellular matrix components, these vectors accumulate at sites of tumor development with newly exposed collagens. When the matrix-targeted retroviral vector expressing dominant mutant cyclin G1 was administered by portal vein infusion, vector particles accumulated in the angiogenic tumor vasculature within 1 hour of infusion. These vectors, Rexin-G, transduced tumor cells with high efficiency and reduced the volume of tumor [23]. In clinical studies, Rexin-G showed significant anti-tumor activities in breast, colon, lung, skin, muscle, pancreatic, and bone cancers [25, 26]. Rexin-G was granted Orphan Drug Status by the US FDA in 2008.

Targeting retroviral delivery to quiescent interleukin-2 (IL-2)-dependent cells was also reported [27]. In this report, chimeric amphotropic MLV envelope glycoprotein fused with IL-2 was used for a direct binding of the viral particles to the IL-2 receptors expressed on G0/G1 arrested cells, resulting in a transient stimulation of cell proliferation. Subsequent viral entry was mediated by unmodified envelope proteins co-expressed on the same virus particles. A 34-fold increase in transduction efficiency was observed with this method. Additionally, targeting efforts for T cells [28], and other cancer cells [29, 30] were reported. For the specific transduction of HIV-envelope expressing cells, envelope pseudotyping was used to create hybrid CD4/CXCR4 receptors for MLV retrovirus [31, 32] and lentivirus [31, 32], In order to improve transduction efficiency frequently observed to be low for the targeted retroviral vectors, binding defective but fusion competent hemagglutinin (HA) protein has also been tried [33].

## LOCAL DELIVERY

If local delivery of retroviral vectors is available for an effective treatment of a disease, it will be generally safer than systemic delivery in terms of toxicology and long term side effects. A number of studies have shown the efficacy and safety of locally delivered retroviral vectors. A retroviral vector expressing antiproliferative dominant negative mutant cyclin G1 (dnG1) was successfully used for the prevention of eximer laser-induced corneal haze [34]. Biodistribution study after the treatment of surgically induced rabbits with eye drops containing dnG1 retroviral vectors showed no evidence of vector dissemination in non-target organs. Localized delivery of lentiviral vectors into the substantia nigra of adult rats has also been tried [35]. In a phase I clinical trial for direct intratumoral injection of interferon-γ retroviral vectors in advanced melanoma patients, viral injection was well tolerated and no toxicity was reported [36]. This suggests that the direct injection approach is feasible for treating solid tumors with retroviral vectors.

In terms of potential problems associated with concomitant transduction of surrounding non-target cells, *ex vivo* cell-based gene therapy with local delivery can be a better choice, if extra time and expenses are tolerated. As an example, an *ex vivo* cell-mediated gene therapy has been performed successfully for the treatment of artificially induced hyaline cartilage damage in animals, by injecting *TGF-β*1-retrovirus transduced fibroblasts into the knee joints [37, 38]. Cancer regressions were reported after the transfer of genetically engineered lymphocytes [39]. Autologous lymphocytes from peripheral blood using retroviruses that encode T cell receptors to specific tumor-associated antigens were transferred to patients. Transduced normal peripheral blood cells were converted into cells which specifically recognized and destroyed cells from corresponding cancers.

## INTEGRATION OF RETROVIRUS INTO THE HOST CHROMOSOME

In the *ex-vivo* gene therapy trials to treat the rare immune deficiency disorder X-SCID, patients were treated with autologous hematopoietic stem cells transduced with a recombinant retrovirus expressing the common gamma chain (γ_c_) of interleukin receptor [4]. Although, nine out of eleven treated children showed dramatic improvements with almost fully restored immune systems, four of the nine cured patients developed leukemia (T-cell acute lymphoblastic leukemia; T-ALL) between 3 and 6 years after the treatment in France. A copy of the vector DNA was found in the first intron of the growth-promoting *LMO2* gene of the leukemic clones in patient 4, and approximately 3 kb upstream of the first exon of the same gene in patient 5 [1-3]. In patient 10, there was another insertion near the proto-oncogene *BMI1* in addition to the first insertion near *LMO2. *In patient 7, blast cells showed an insertion near a third proto-oncogene CCND2 [40]. Another case of leukemogenesis was reported in a separate X-SCID study conducted in United Kingdom [6]. In addition to the integration of vector in the upstream of *LMO2* gene, gain-of-function mutation in NOTCH1, deletion of tumor suppressor gene locus cyclin-dependent kinase 2A (CDKN2A), and translocation of the TCR-b region to the STIL-TAL1 locus were found. Two cases of myelodysplasia were reported in a clinical trial for X-linked chronic granulomatous disease (X-CGD) [7]. Both patients showed an insertional activation of ecotropic viral integration site 1 (EVI1) and monosomy 7. LMO2 is a LIM domain transcription regulator involved in hematopoiesis [41] and is reported to be activated in T cell leukemia by chromosomal translocation [42]. LMO2 is suggested to reactivate a hematopoietic stem cell (HSC) specific transcriptional program [43]. Long-term thymocyte self-renewal due to the over-expression of LMO2 could be the cause of T cell leukemia. The BMI1 gene was known to regulate stem cell proliferation [44, 45]. MMI1 is suspected to contribute the leukemic cell proliferation with LMO2. Massive and sustained expression of CCND2 was detected in the CCND2-rearranged T-ALL, compared to the down-regulation during the progression from the early stages of normal human T-cell [46]. Over-expression of CCND2 in one of the patients could be one of the oncogenic transition mechanisms to T-ALL.

Current U.S. Food and Drug Administration (FDA) perspective on X-SCID clinical trials is that gamma-retroviral vectors can be used in clinical trials to treat X-SCID under the following conditions: when previous hematopoietic stem cell/bone marrow transplantation is failed or there is no reasonable alternative therapy [47]. Although, clinical trials are allowed to proceed for other clinical indications, investigators and patients are required to be informed with strong and clear communication of risks. 

The general consensus of the experts of the review boards in the US and other countries is that the benefits might outweigh the risks in most SCID-related retroviral gene therapy trials. In a Recombinant DNA Advisory Committee (RAC) meeting held on March 14, 2007, it is recommended for all integrating vectors to test for vector sequences every 6 months first 5 years and test yearly next ten years or until no vector is detected. When at least 1% of surrogate cells have detectable vector, pattern of vector integration site should be assessed. In case persistent monoclonality or vector integration near or within locus known to have oncogenic activity, additional monitoring is recommended.

## TARGETING RETROVIRAL INTEGRATION; MLV VS HIV

After entering the host cell, a single-stranded retroviral RNA genome is released into the cytoplasm and converted into a double-stranded DNA by virus-encoded reverse transcriptase. The viral DNA then forms a large nucleoprotein structure, termed pre-integration complex, containing proteins necessary for nuclear localization and insertion of viral DNA into the host genome. Although the protein components and the exact mechanism of action of the complex is still not completely understood, it has been demonstrated that viral integrase (IN) catalyzes the key DNA cutting and joining reactions for inserting viral DNA into the host genome [48, 49].

Retroviral integration is not a completely random process but favors promoters and enhancer regions while lentiviral vectors integrate more randomly throughout the entire gene [50, 51]. Due to the differences of insertional preferences between two vectors, it was considered that HIV based gene therapy is safer than MLV based one. However, recent result from a gene transfer study of β-thalassemia Major and Sickle Cell anemia conducted in France suggests a different story [52]. Office of Biotechnology Activities of National Institutes of Health (NIH) published a letter in June, 2009 that a relative clonal dominance was detected in a subject with β-thalassemia Major, two years after the treatment. The vector used in the study was a self-inactivating (SIN) HIV-1 derived lentivirus which contains the gene for β-globin under the control of the β-globin promoter. Clonal populations share an integration site in *HMGA2* gene. This incident raised a question about whether the use of lentiviral and modified SIN retroviral vectors containing insulators can decrease the risk of insertional mutagenesis in hematopoietic stem cells.

There are controversial reports about the function of viral proteins including integrase. Swapping the integrase between closely related viruses showed a change in the integration pattern [53, 54]. However, changing of gag, env, and pol genes of MLV with those from subgroup C Feline Leukemia Virus (FeLV-C) did not alter the basic integration profile [55]. Elucidating the mechanisms of integration and establishing the database for preferred integration sites could permit a better prediction of the integration sites of retroviral vectors. This may eventually lead to the development of retroviral vectors capable of integration site selection in the host cell chromosome, providing the ultimate solution to the problems of insertional mutagenesis. 

There have been attempts to target retroviral integration to pre-selected locations of the host genome by fusing viral integrase with sequence-specific DNA binding domains obtained from phage lambda repressor, bacterial LexA, or a zinc finger protein zif268 [56-58]. However, these trials show only a limited success, as the specificity of integration was only partially altered. In a separate experiment, bovine leukemia virus (BLV) integrase was used for site-specific integration of naked DNA to the pre-integrated integrase recognition sequence of mouse genome [59]. Similarly, site-specific integration of naked DNA into human chromosome 8 has been attempted with limited success using modified phage φC-31 integrase [60]. In this study, enhanced sequence specificity and increased integrase efficiency was achieved through a directed evolution strategy. It is clear that concentrated efforts are required in defining the precise mechanism of action of the retroviral pre-integration complex and in designing modified integrases with sequence-specific integration capability. The latter may be accomplished either by rational modification of the protein or by using the directed evolution approach [61]. One example of rational modification is fusing integrase with synthetic zinc finger motifs with defined sequence specificities [58, 62, 63]. Directed evolution utilizes error-prone PCR-driven mutagenesis, recombination, or DNA shuffling, combined with a high throughput screening for the selection of modified proteins with significantly improved function. The newly developed integrases should also maintain the ability to form a pre-integration complex with a high-level of infection capability. 

Generation of integration deficient lentiviral vectors was also reported [64, 65]. The vector showed durable transcription of transgenes in certain mitotic cell lineages, but non-integrated viruses were lost during cell division.

## INSULATORS TO PREVENT POSITIONAL EFFECTS AND INSERTIONAL ONCOGENE ACTIVATION 

Retroviruses are often susceptible to positional effects and transcriptional silencing depending on the site of integration in the chromosome [66]. In order to overcome positional silencing effect, chromatin insulators have been used in retroviral vectors. Chromatin insulators are believed to form expression boundaries [67, 68] and can block positive and negative positional effects at the site of integration when they flank a transgene [69-71]. They prevent interferences between promoters and enhancers of adjacent genes [72]. As an example, when a 1.2 kb chromatin insulator obtained from the chicken β-globin locus control region hypersensitive site 4 (cHS4) was inserted in the retrovirus 3’ LTR, protection of the positional effects was observed either from transduced cultured cells and from mice transplanted with transduced marrow cells [73]. Similarly, cHS4 insulator used with gamma-globin expression cassette increased the likelihood of stable gamma-globin expression nearly 10-fold, allowing for the expression at the therapeutic range for treating sickle cell anemia and beta thalassemia in mouse bone marrow transplantation models [74]. A part of full length cHS4 (650 bp) was confirmed to work for the practical purpose [75]. Insertion of a cHS4 in SIN lentiviral vectors resulted in higher and less variable expression of human ß-globin [76, 77].

Because sequences in the 3’ LTR of retroviral vectors are copied to the 5’ LTR during the processing of viral genome into the provirus, if an insulator is inserted in the 3’ LTR of the recombinant vector, a barrier of insulators will be formed surrounding the transgene Fig. (**1A**). In addition, insulators are inserted in the place of the U3 region of the 3’ LTR, which is the viral enhancer region. These vectors will become SIN in the proviral form, as there is no viral enhancer required to produce replication competent retrovirus. Therefore, insulator containing retroviral vectors will be less prone to silencing of the transgene expression as a result of chromosome positional effect [73], and at the same time, will have less chance of causing aberrant induction of host genes near the site of incorporation as it has no viral enhancer. Boundaries formed by insulators will also prevent the influence of an internal heterologous enhancer used to drive transgene expression on the transcription of nearby host genes, although it has yet to be experimentally proven.

Although retroviral insertion can cause either a disruption or an abnormal activation of host genes, the latter is a primary concern in terms of oncogenesis. This is because, in most cases, retroviral insertion will occur in only one allele of the host genome leaving the other locus intact, and insertional disruption of the host gene will become problematic only in rare cases where haplo-insufficiency is phenotypically relevant for oncogenic transformation. Thus, although chromatin insulators cannot prevent host gene disruption by retroviral insertion, the benefits associated with the use of insulators preventing unwanted activation of host genes will be rather significant. 

The efficiency of insulator function is, however, dependent on several factors including topological constraints, cell types, and the state of cell differentiation [78, 79]. Also the size limitations of retroviral vectors should be considered. A 265 bp sea urchin insulator termed *sns* (silencing nucleoprotein structure) was found to be effective for insulator function in human cells, and thus may be useful in retroviral vectors [80, 81]. In a recent report, anti-repressor elements were identified by screening a library of human genomic DNA fragments between 500 and 2,000 bp, based on their ability to relieve LexA-dependent transcription repression [82]. These elements can confer high and stable transgene expression in mammalian cells when they were used to flank the transgene, suggesting that they play a similar role as insulators. 

Scaffold (or matrix) attachment region (SAR) is another DNA sequence element believed to play an important role in defining boundaries of independent chromatin domains [83, 84]. SARs bind to the nuclear scaffold or nuclear matrix with high affinity and are proposed to form chromosomal loops [85]. SARs have been used in retroviral vectors with an enhancement of transgene expression in several different cell types [86, 87]. A report shows that a high-level transgene expression can be achieved from a SIN lentiviral vector containing both the human interferon-beta scaffold attachment region and the chicken β-globin insulator [88]. The proviral form of this vector does not contain HIV-1 U3 region transcriptional regulatory elements and is flanked by the enhancer-blocking β-globin insulators. These observations indicate that the usage of SARs in addition to insulators could significantly improve transgene expression and lower the risk of uncontrolled activation of cellular proto-oncogenes at or near the site of incorporation.

Activation of proto-oncogene may also arise due to the retroviral RNA processing. A strong internal splice acceptor (SA) is recommended after the splice donor (SD) of retroviral vector to reduce the positional effects of inserted retroviral RNA processing on the expression of the transgene as well as the disrupted host gene. Combination of a strong SA, deleting cryptic SD in the transgene [89], using an improved polyadenylation signal [90], the removal of LTR promoter, and the insulator will largely prevent the interactions of the retroviral splice donor with downstream chromosome sequences.

## TRANSCRIPTIONAL TARGETING

Transcriptional targeting using cell type-specific promoters and enhancers can be applied either alone or in combination with targeted transduction to minimize the expression of transduced genes in non-target cells and thereby reducingpotential side effects. Table **2** summarizes examples of cell type-specific promoters and enhancers used for transcriptional targeting in retroviral gene therapy. Promoters of oncogenes overexpressed in the tumor cells can be the targets for tumor specific promoters (e.g. *c-erbB2* and *c-myc*). Tyrosinase promoter was used for the expression of HSV-tk or IL-2 for the treatment of malignant melanomas [91]. Tyrosinase is rate-limiting enzyme for melanin production, which is highly expressed in melanomas. In addition to cell type-specific promoters, inducible or regulatable expression systems can also be used for safety and efficacy. In the case for mammary tumor and prostate cancer, the growth of tumor is hormone dependent. Therefore, using a combined steroid hormone-responsive and cell type specific promoters is an attractive approach for retroviral gene therapy [92]. Additionally, genes that are induced by cancer therapies such as gamma-irradiation or chemotherapy can also be the targets for the regulatory elements [93, 94]. 

Although a number of cell type specific promoters were tested in many trials, the overall efficiency of transcription achieved from cell type specific promoters is relatively weak compared to viral promoters generally used. In a report, hypoxic and cytokine-inducible enhancers, both of which are active in some tumor environments, are combined with endothelial cell-specific E-selectin and VEGF receptor 2 promoters [95] to achieve a maximum possible tumor endothelium-specific transcription. In another report, human α-fetoprotein (AFP) enhancer was combined with a housekeeping gene phosphoglycerate kinase-1 (PGK-1) promoter, to augment the activity of the weak tumor-selective AFP promoter [96]. 

Rat alpha-fetoprotein promoter was used as a cell type-specific promoter for a lentivirally transduced expression in human hepatocarcinoma cells [97]. Replacement of U3 region of the lentiviral LTR with an upstream enhancer (HS2) of the erythroid-specific GATA-1 gene and HIV-1 promoter showed a high level of transgene expression specifically in mature erythroblasts [98]. 

In many cases, the size constraints of retroviral vector limit the use of enhancers, which are generally long in size. Therefore, construction of minimum enhancer/promoter cassettes with strategic combinations of different sequence elements will be required to facilitate the efficacy of gene therapy trials. 

## COEXPRESSION OF A SUICIDAL GENE

Herpes simplex virus thymidine kinase (HSV-tk) has been used for selective destruction of cells in several different settings. When anti-viral prodrug nucleobase analogue ganciclovir (GCV) is applied to HSV-tk expressing cells, GCV is efficiently converted into monophosphate form by HSV-tk, and then into cytotoxic triphosphate derivatives by cellular kinases. Actively dividing cells will be killed as they incorporate the nucleotide derivatives into their genome. In allogeneic bone marrow transplantation (BMT), donor T cells are able to mediate anti-leukemic effects but they can also induce graft-vs-host disease (GvHD), which is often fatal. In an attempt to reduce GvHD while maintaining anti-leukemic effect, scientists have retrovirally transduced HSV-tk to donor T-cells before being used in animal myeloablative BMT trials [108]. At first, the donor T-cells helped to eliminate residual malignant leukemic cells, but when signs of GvHD development were noticed, proliferating donor T-cells were rapidly destroyed by treating the animals with ganciclovir. When this strategy was used in a human trial, three out of eight patients treated with donor lymphocytes transduced with HSV-TK gene could be effectively controlled by ganciclovir-induced elimination of the transduced cells when they developed GvHD 12 months after transduction [109].

Similarly, retroviral vectors can be designed to co-express HSV-tk suicide gene to be used as a safety switch, in addition to a therapeutic gene. If abnormal growth of transduced cells is observed such as the cases in the X-SCID, treatment with ganciclovir can eliminate all the transduced cells theoretically. However, constitutive expression of HSV-tk can also induce the death of neighboring uninfected cells by the bystander effects when ganciclovir is administered. In order to minimize unwanted side effects due to bystander effects, the use of cell type-specific or inducible promoter for the expression of HSV-tk or the use of other pro-apoptotic genes with a minimum bystander effect may be advantageous. As an example, lentivirally transduced expression unit containing the rat alpha-fetoprotein promoter was used to restrict the HSV-tk induced GCV sensitivity to human hepatocarcinoma cells [97]. 

On the other hand, retroviral vectors expressing HSV-tk have been used in antitumor treatment trials [110-112]. In this case, maximum “bystander effect” is required to kill neighboring uninfected cells as well as infected cells. The results of tumor treatment with HSV-tk expressing retroviral vectors were, however, not fully successful due to low infection efficiency and weak bystander effects. 

One potential obstacle for co-expressing HSV-tk suicide gene as a safety switch in addition to the therapeutic gene however, is the limited insert size constraint of the retroviral vector. In cell-based *ex-vivo* gene therapy using the clonally-derived cells, selection of single clones, transduced with two separate retroviral vectors harboring the HSV-tk gene and the therapeutic gene in each vector, could be a solution for the problem of size restriction. 

## AVOIDING REPLICATION COMPETENT RETROVIRUS (RCR)

Generation of RCR remains as a potential safety issue in retroviral gene therapy. Retroviral vectors transfected into a packaging cell line can produce RCR by recombination processes between homologous sequences of the retroviral vector DNA and the *gag, pol, *and *env* coding sequences in the packaging systems. In order to lower the chances for recombination, both minimizing the homologous sequences and physically separating genes for *gag, pol *and *env* into two different expression cassettes, have become standard practices. However, residual *gag, pol, *and *env* coding sequences are frequently included in these vectors in an attempt to increase transduction efficiency and viral titer. Therefore, remaining part of *gag, pol, *and *env *gene is raising a concern for RCR generation. There is a report that a complete removal of residual coding sequences for *gag, pol, *and *env* genes did not show any detrimental effect on viral transduction efficiency, and it also reduced the chance of RCR generation [113]. The same concept was also applied to lentiviral vectors. To avoid the production of replication competent lentivirus (RCL), the components required for the production of lentivirus were divided into at least three parts: vector plasmid which contains the gene of interest and the minimal *cis*-acting element of HIV; packaging plasmid which has all HIV viral genes except the *env* gene; envelope proteins which were provided from a plasmid containing the envelope gene via co-transfection. Typically, the Glycoprotein from vesicular stomatitis virus (VSV-G) is used as an envelope gene [114]. One of the safety concerns specific to HIV virus is the recombination between vector sequences and endogenous HIV sequences in HIV positive patients. In spite of the large deletion of endogenous retroviral sequences, the possibility of recombination and mobilization cannot be overlooked. Stable lentivirus producer cell line has been tried due to the higher probability of recombination during the packaging process through transient transfection is much higher than that of the stable producer cell lines [115, 116]. Due to the concerns of using HIV in humans, researchers are developing non-human lentiviral vector systems. Simian immunodeficiency virus (SIV), Feline immunodeficiency virus (FIV), and Equine immunodeficiency virus (EIAV) are some of them [117-119]. However, the safety features of non-primate lentiviruses in humans have yet to be determined [120].

SIN retroviral vector was developed by introducing a deletion in the U3 region of the 3’ LTR which contains all the enhancer and promoter activities of the viral vector Fig. (**1B**) [121]. No active viral particle is arising from SIN vectors, because the 5’LTR carrying the same deletion in the chromosome is not capable of inducing transcription for the production of packagable RNAs. An additional advantage of the SIN vector is the minimal chance of LTR-mediated insertional activation of proto-oncogene near the site of insertion. SIN vector approach has been tested more extensively in lentiviruses [122]. Although SIN vector was considered as an ideal vector, a SIN vector mobilization was detected with a very low level [116]. Additionally, one of the drawbacks of SIN retroviral vectors is the low transcriptional activity of the internal promoter compared to the viral LTR in a number of different cell types. Improvements in the design of the internal promoter/enhancer are required to overcome this obstacle. 

Split-intron retroviral vector has been shown to enhance expression of an inserted gene and safety was improved [90]. A strong synthetic splice donor (SD) site and a splice acceptor (SA) site were inserted between U3 and R of the 3’ LTR and downstream of the packaging signal, respectively. During the reverse transcription process, the strong synthetic splice donor site introduced in the 3’ LTR is copied into 5’LTR, and theoretically all the transcripts made in the transduced cells are spliced and the packaging signal is removed Fig. (**1C**). Therefore, the possibility of producing RCR is greatly reduced. 

Finally, activation of proto-oncogene may arise due to aberrant retroviral RNA processing. A strong internal SA is recommended after SD site of retroviral vector to prevent a generation of aberrant read through transcripts containing both the transgene and a portion of the disrupted host gene. Combination of strong SA, deleting cryptic SD in the transgene [89], an improved polyadenylation signal [123], removal of LTR promoter, and the presence of an insulator will largely prevent the interactions of the retroviral splice donor with downstream chromosome sequences.

## CONCLUDING REMARKS

Applications of gene therapy protocols have been continuously expanded to wide variety of acquired and inherited diseases, such as cancer, SCID, and other life threatening diseases. Retroviral gene therapy approaches for the treatment of these diseases have to address safety issues. Targeted infection, local delivery, targeted retroviral insertion, insulators, transcriptional targeting, co-transduction with a suicidal gene, and SIN vectors were suggested as possible solutions for the risks of retroviral gene therapy. Some of these precautions can also be applied to gene therapy protocols using other viral and non-viral vector systems. One major immediate concern in terms of retroviral gene therapy, as revealed by the X-SCID case, is insertional oncogenesis. Several approaches to decrease the possibility of insertional oncogenesis were considered in depth. In addition to the widely used retroviral systems that were discussed above, foamy viruses may be used as a safe and efficient means of targeting non-dividing cells [124, 125]. Foamy viruses are known to have a broad host range, without causing any disease and persist in infected humans [126-128]. In the case of *ex vivo* cell-based gene therapy, transduced cells could be pre-screened to select for clones with the insertion of the transgene only at a desirable site of the chromosome, which can minimize the chances for insertional oncogenesis. Orthopedic indication is one of the most promising areas of gene therapy in spite of the non-lethal conditions [37, 129-135]. 

The incident from a gene transfer study of β-thalassemia Major in France showed us that there is no guaranteed way to safe gene therapy. Pursuing prudent designing of vectors and monitoring adverse effects in patients are proper directions. Additionally, selection of patient specific method is recommended for each patient based on the risk versus benefit.

## Figures and Tables

**Fig. (1):**
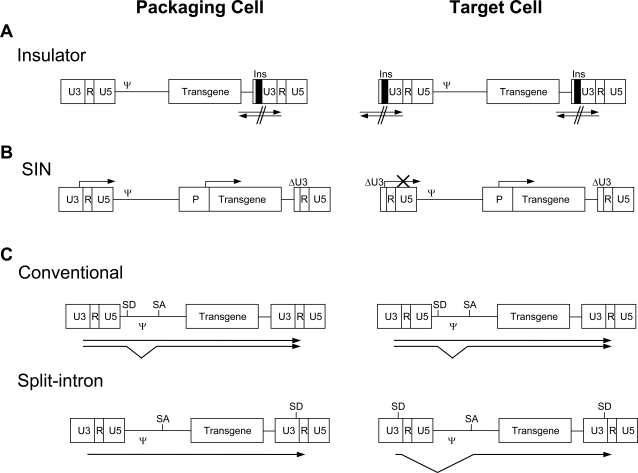
Designs of the improved retroviral vectors. (**A**) Insulator located in U3 of 3’LTR is copied to 5’LTR in target cell. Insulators located in the both ends of vector DNA blocks the cross activation between retroviral DNA and the chromosomal DNA. (**B**) Due to the duplication of U3-deleted LTR of the SIN vector in target cell, the proviral form does not contain viral enhancer any more, and thus require an internal promoter for the expression of the transgene. Arrows show the transcription start. P, internal promoter; ΔU3, deletion of U3 sequence. (**C**) Comparison of splicing in conventional and split-intron vectors. Arrows show transcripts from the vector DNA. SD, Splice Donor site; SA, Splice Acceptor site.

**Table 1:** Retroviral Vectors for Targeted Infection

Modification	Target Cell	References
EGF-MMP cleavable linker chimeric env	Cancer invasion, angiogenesis, inflammation	[16, 17, 20]
IL-2 chimeric env	IL-2 R	[27]
EGF chimeric env	EGFR	[15]
SCF-Factor Xa chimeric env	Stem cell (Kit)	[19]
vWF (collagen binding) chimeric env	Cancer (collagen expressing); vascular lesion	[23, 24]
Single-chain variable fragmented antibody (scFv) for EGFRvIII	Cancer (brain, breast, lung, ovary)	[29]
scFv for HMWMAA	Cancer	[22]
scFv from phage display	T cell	[28]
scFv for Carcino embryonic antigen (CEA)	Cancer	[30]
Receptor pseudotyping (CD4 and CXCR4)	HIV-1 infected cell	[31, 32]

**Table 2:** Cell Type-Specific Promoters and Enhancers for Transcriptional Targeting in Retroviral Gene Therapy

Promoter	Target Cell/Tissue	Transgene	References
PEPCK promoter	Hepatocyte	Neo, bovine growth hormone	[99]
hAAT promoter	Hepatocyte	Alpha I antitrypsin	[100]
MMTV-LTR	Mammary gland	TNF-α	[92]
MCK promoter	Muscle	β-galactosidase, dystrophin minigene	[101]
AFP promoter	Cancer: Hepatocellular carcinomas	HSV-tk, VZV-tk	[102]
Tyrosine promoter	Cancer: Melanomas	HSV-tk, IL-2	[91]
Col1a1 promoter	Bone	β-geo (β-gal, neo fusion)	[103]
HSP70 promoter	Cancer	Dominant negative IGF-IR	[104]
WAP promoter	Cancer: Mammary	β-galactosidase	[105]
ppET1 promoter	Cancer: Endothelium	β-galactosidase	[106]
AFP enhancer; PGK promoter	Cancer: Hepatocellular carcinomas	HSV-tk	[96]
HRE, PGK-1 enhancer; E-selectin, KDR promoter	Cancer: Endothelium	TNF-α, luciferase	[95]
HRE enhancer; AFP promoter	Cancer: Hepatocellular carcinomas	HSV-tk, luciferase	[107]
Rat alpha-fetoprotein	Human hepatocarcinoma cell	HSV-tk, luciferase	[97]
HS2 of erythroid-specific GATA-1 gene; HIV-1 promoter	Mature erythroblasts	GFP	[98]
